# Real-world effectiveness and predictors of treatment failure for eravacycline in patients with *Acinetobacter baumannii* or *Klebsiella pneumoniae* infections: a multicenter retrospective analysis

**DOI:** 10.3389/fcimb.2026.1746200

**Published:** 2026-03-04

**Authors:** Yi Li, Qiaolian Yi, Menglan Zhou, Minya Lu, Yingchun Xu

**Affiliations:** 1 Department of Clinical Laboratory, Peking Union Medical College Hospital, Chinese Academy of Medical Sciences and Peking Union Medical College, Beijing, China; 2 State Key Laboratory of Complex Severe and Rare Diseases, Peking Union Medical College Hospital, Chinese Academy of Medical Sciences and Peking Union Medical College, Beijing, China

**Keywords:** *Acinetobacter baumannii*, antimicrobial stewardship, carbapenem, eravacycline, *Klebsiella pneumoniae*

## Abstract

**Introduction:**

Eravacycline (ERV) is a novel synthetic fluorocycline antibiotic with broad-spectrum antibacterial efficacy against pathogens. This study aimed to investigate the clinical effectiveness of eravacycline and its correlation with minimum inhibitory concentrations (MICs) against infections caused by *Acinetobacter baumannii* or *Klebsiella pneumoniae*.

**Methods:**

This retrospective multicenter study investigated the real-world use of ERV in 1,796 adults with infection caused by *A. baumannii* or *K. pneumoniae* in China. Antimicrobial susceptibility of strains and laboratory test results during treatment were analyzed. Microbiological and clinical outcomes were assessed at the end of treatment and day 30.

**Results:**

The overall susceptibility rate to ERV was 96.0% (1,027/1,070), and around 98% of carbapenem-resistant isolates were susceptible to ERV. ERV had a 4-fold lower MIC_90_ than tigecycline against both pathogens. At end of treatment, treatment success (microbiological eradication or clinical resolution) occurred in 82.6% (1,483/1,796) of the cohort with microbiological eradication achieved 76.1% (789/1,037). At day 30, infection cure was achieved in 83.57% (1,501/1,796) of the cases. Different ERV regimens (monotherapy or concomitant therapy) had no influence on the treatment and 30-day clinical outcomes. Multivariable analysis identified that elevated C-reactive protein (CRP) levels during treatment, bloodstream infection, sepsis and specific clinical interventions (e.g., central venous catheterization) were independent predictors of treatment failure.

**Conclusions:**

The study highlights ERV’s utility in infection of *A. baumannii* or *K. pneumoniae*, especially carbapenem-resistant strains.

## Introduction

Eravacycline (ERV) is the first fully synthetic tetracycline (TET) compound which demonstrates robust activity against a wide range of pathogens, encompassing aerobic and anaerobic Gram-negative and Gram-positive strains, excluding *Pseudomonas aeruginosa* and *Burkholderia cenocepacia* (Sutcliffe et al., 2013). Similar to other TETs, it inhibits protein synthesis through binding to the 30S ribosomal subunit. ERV has been approved by the Food and Drug Administration (FDA) in 2018 for the treatment of complicated intra-abdominal infections in patients aged 18 and above (Alosaimy et al., 2020a).

Most existing prospective clinical trials and meta-analysis have demonstrated similar efficacy of ERV to carbapenems in the treatment of complicated intra-abdominal infections (cIAIs) (Solomkin et al., 2017; Lan et al., 2019; Solomkin et al., 2019; Alosaimy et al., 2020b; Eljaaly et al., 2021). Clinical trials usually limited by sample size and have exclusively focused on the infection site rather than the causative organism. Infections caused by carbapenem-resistant *Enterobacterales* (CRE) and carbapenem-resistant *A. baumannii* (CRAB) represent formidable challenges in hospital settings. *A. baumannii* is responsible for a spectrum of nosocomial infections, most notably ventilator-associated pneumonia and central-line-associated bloodstream infections (Marí-Almirall et al., 2017; Harding et al., 2018). *K. pneumoniae* is an opportunistic pathogen of grave clinical concern, characterized by a convergence of multidrug resistance, high virulence, and efficient intra- and interspecies transmissibility (Lei et al., 2024). Among Gram-negative pathogens, only the MIC breakpoints of ERV against *Escherichia coli* were provided. Clinical evaluation of ERV against *A. baumannii* or *K. pneumoniae*, especially carbapenem-resistant strains are lacking. More importantly, most clinical trials were conducted in western countries and clinical evaluation of ERV on Asian population is blank. In China, eravacycline is approved for the treatment of complicated intra-abdominal infections (cIAI) in adults. This study reflects its real-world use across various infection sites, which includes many off-label uses reflective of clinical practice for difficult-to-treat infections.

On January 29, 2024, the clinical breakpoints of ERV against *A. baumannii* and *K. pneumoniae* was approved by the Committee of the National Health Commission on Antimicrobial Susceptibility Testing and Standard Research (ChinaCAST) in China. Supported by data from multicenter of China, the present study analyzed 1,796 cases who were treated with ERV against infection caused by *A. baumannii* or *K. pneumoniae* from Sep 2023 to Sep 2024. We aimed to explore the clinical and microbiological outcomes among patients treated with ERV in the real-world setting amid Chinese population.

## Materials and methods

### Study design and patient population

The present study was conducted among hospitalized patients (>18-years-old) receipt of ≥72 consecutive hours of ERV therapy for infection in participating hospitals from 21 provinces of China during September 2023 to September 2024. The data for this multicenter study were systematically obtained using a standardized “Eravacycline Clinical Application Evaluation Data Collection Form”, which was uniformly used by reporting physicians across all participating centers. This form was specifically designed for this study to ensure consistent and comprehensive data capture across all sites, encompassing key elements such as patient demographics, infection details, microbiological results, treatment regimens, and clinical outcomes.

Patients with multiple pathogens were excluded to avoid confounding variables, ensuring that the observed clinical and microbiological outcomes could be attributed specifically to the treatment of *A. baumannii* or *K. pneumoniae*. A total of 1,796 adult patients with *A. baumannii* (n=1,214) or *K. pneumoniae* (n=582) infections were included. Infection was defined by physicians according to positive culture, clinical signs, laboratory test results and imaging evidence. To differentiate infection from colonization, we required not only a positive culture but also corresponding clinical symptoms (e.g., fever, purulent sputum), elevated inflammatory markers, and/or radiological evidence of new infiltrates. The following patient characteristics were collected: demographics, underlying conditions, immunosuppressive regimen, recent surgery, medical ventilation, laboratory test results (WBC count, neutrophil count, CRP and PCT) before, at the 3^rd^ day and the end of treatment, clinical outcomes at the end of treatment, clinical outcomes at day 30. Clinical data were systematically collected by thoroughly reviewing the medical charts that were implemented in the centralized medical software of each hospital. The following microbiological characteristics were recorded: causal pathogen(s), antimicrobial susceptibility testing (AST) by broth microdilution and/or disk diffusion, and microbiological outcomes at the end of treatment (eradication, persistence, relapse with another pathogen). Finally, ERV treatment characteristics were recorded: dose, duration of intravenous infusion and side effects. ChinaCAST recommends that MIC ≤ 1mg/L by broth microdilution or disk diffusion zone diameters ≥15 mm to be considered as susceptible. The MIC values reported in this study were determined quantitatively using the broth microdilution method. Concomitant therapy was defined as any therapy used in conjunction with eravacycline for ≥48 continuous hours for the primary organism that eravacycline therapy was used for.

This study was conducted in accordance with the Declaration of Helsinki. Given the retrospective observational nature of the study and the absence of any modification to patient management, the need for informed consent was waived by the ethics committee. All data were anonymized and kept confidential. The study protocol was approved by the Ethics Committee of Peking Union Medical College Hospital (Protocol code I-23ZM0067).

### Endpoint

The primary objective consisted of determining the microbiological and clinical outcome at the end of treatment. Microbiological outcomes including eradication, persistence and relapse with another pathogen. For cases who were unable to collect specimen for bacteria culture, clinical outcomes at the end of treatment were analyzed which included clinical resolution (clinical success): defined as complete resolution or significant improvement of clinical signs and symptoms of the index infection; clinical failure: defined as persistence or worsening of signs and symptoms, or the requirement of additional rescue therapy.

Secondary objectives were to determine the clinical outcome at day 30, including infection cure (resolution of all signs and symptoms, normalization of laboratory findings, and confirmed/presumed pathogen eradication), relapse (recurrence of clinical manifestations with abnormal laboratory findings), attributable mortality (death directly related to the index infection) and unattributable mortality (death from unrelated causes during follow-up).

### Statistical analyses

Medians and IQRs are presented for continuous variables. Categorical variables were expressed as % (m/n) and examined using χ^2^/Fisher’s exact test. *P* value < 0.05 was considered statistically significant. Multivariable logistic regression models were constructed to identify independent factors associated with the primary composite outcome of treatment failure and with 30-day overall mortality. Variables with a P-value < 0.1 in univariate analysis were included in the initial models. A stepwise selection method was used to retain significant predictors in the final models. Results are presented as adjusted odds ratios (ORs) with 95% confidence intervals (CIs). Statistical analyses were performed and graphs were plotted using R (4.2.1) (https://cran.r-project.org).

## Results

### Demographics, clinical course and treatment characteristics

A total of 1,796 adult patients (aged over 18 years) from 21 Chinese provinces received ≥72 hours of eravacycline for *A. baumannii* (n=1,214) or *K. pneumoniae* (n=582) infections (**Table 1**). The cohort exhibited male predominance (sex ratio 1.98:1) with a median age of 62 years (IQR 50–74). No intergroup difference was observed in median treatment duration (8 days). Patients with *A. baumannii* infections had higher rates of ICU admission (63.8% vs 44.2%; *P* < 0.001) and mechanical ventilation (60.0% vs 46.4%; *P* < 0.001), whereas *K. pneumoniae* infections were associated with greater hematological comorbidities (25.8% vs 11.0%; *P* < 0.001) and neutropenia (20.6% vs 10.7%; *P* < 0.001).

**Table 1:** Characteristics and clinical information of patients.

Characteristics	Patients infected with *A. baumannii*	Patients infected with *K. pneumonia*	Total	*P*
Number	1,214	582	1,796	
Median age (IQR)	63 (50-75)	60 (48-72)	62 (50-74)	0.009
Sex ratio (M/F)	2.1	1.93	1.98	0.482
Departments				
ICU	63.76% (774)	44.16% (257)	57.41% (1031)	<0.001
Hematology	6.43% (78)	20.79% (121)	11.08% (199)	<0.001
Transplant	8.57% (104)	5.67% (33)	7.63% (137)	0.039
Respiratory	3.95% (48)	4.47% (26)	4.12% (74)	0.7
Emergency	2.31% (28)	3.61% (21)	2.73% (49)	0.153
Infection	1.89% (23)	2.41% (14)	2.06% (37)	0.592
Others	13.1% (159)	18.9% (110)	14.98% (269)	0.002
Specimen type				
Sputum	53.79% (653)	50.17% (292)	52.62% (945)	0.166
BALF	31.47% (382)	28.52% (166)	30.51% (548)	0.225
Blood	7.33% (89)	11.68% (68)	8.74% (157)	0.003
Peritoneal fluid	7.41% (90)	9.62% (56)	8.13% (146)	0.131
Infection Classification				
Pneumonia	91.7% (1113)	86.8% (505)	90.1% (1618)	0.001
Lung abscess	3.1% (38)	2.1% (12)	2.8% (50)	0.256
Intra-abdominal infection	13.7% (166)	16.5% (96)	14.6% (262)	0.13
Bloodstream infection	12.7% (154)	16.7% (97)	14.0% (251)	0.027
Urinary tract infection	2.1% (25)	3.3% (19)	2.4% (44)	0.167
Central nervous system infection	0.6% (7)	0.2% (1)	0.4% (8)	0.45
Underlying Diseases and Comorbidities				
Pulmonary diseases	31.5% (382)	23.0% (134)	28.7% (516)	<0.001
Solid tumors	9.6% (116)	10.8% (63)	10.0% (179)	0.449
Hematological diseases	11.0% (133)	25.8% (150)	15.8% (283)	<0.001
Cardiovascular diseases	32.7% (397)	30.4% (177)	32.0% (574)	0.358
Neurological diseases	17.4% (211)	18.7% (109)	17.8% (320)	0.527
Renal diseases	22.3% (271)	20.3% (118)	21.7% (389)	0.355
Diabetes	21.1% (256)	25.8% (150)	22.6% (406)	0.031
AIDS	2.6% (32)	3.3% (19)	2.8% (51)	0.549
Rheumatic and immunological diseases	2.5% (30)	2.7% (16)	2.6% (46)	0.85
History of splenectomy	2.0% (24)	0.9% (5)	1.6% (29)	0.119
Hemodialysis	12.0% (146)	10.3% (60)	11.5% (206)	0.322
Recent chemotherapy/radiotherapy	9.3% (113)	21.0% (122)	13.1% (235)	<0.001
Mechanical ventilation	60.0% (729)	46.4% (270)	55.6% (999)	<0.001
Sepsis	30.6% (371)	29.4% (171)	30.2% (542)	0.65
Neutropenia	10.7% (130)	20.6% (120)	13.9% (250)	<0.001
Recent surgery	33.9% (412)	29.2% (170)	32.4% (582)	0.051
Post-transplantation status	17.2% (209)	15.8% (92)	16.8% (301)	0.496
Long-term Corticosteroid/immunosuppressant use	23.6% (286)	24.6% (143)	23.9% (429)	0.681
Invasive Devices and Procedures				
Arterial catheterization	40.0% (485)	27.0% (157)	35.7% (642)	<0.001
Central venous catheterization	61.1% (742)	57.0% (332)	59.8% (1074)	0.11
Endotracheal intubation	64.1% (778)	48.6% (283)	59.1% (1061)	<0.001
Tracheostomy	40.7% (494)	35.1% (204)	38.9% (698)	0.025
Foley catheter	61.7% (749)	47.9% (279)	57.2% (1028)	<0.001
Nasogastric tube	63.1% (766)	52.1% (303)	59.5% (1069)	<0.001
Dialysis therapy	18.9% (230)	15.1% (88)	17.7% (318)	0.055
Other indwelling catheters	15.0% (182)	10.5% (61)	13.5% (243)	0.011
Treatment characteristics				
Treatment duration of ERV (IQR)	8 (6-11)	8 (6-12)	8 (6-11)	0.066
Active therapy before ERV ^a^	40.2% (488)	48.6% (283)	42.9% (771)	<0.001
Meropenem	19.4% (236)	22.9% (133)	20.5% (369)	0.107
Tigecycline	11.3% (137)	10.7% (62)	11.1% (199)	0.75
Cefoperazone-Sulbactam	10.0% (122)	9.5% (55)	9.9% (177)	0.753
Imipenem	4.5% (55)	6.7% (39)	5.2% (94)	0.069
Ceftazidime-Avibactam	3.0% (37)	8.1% (47)	4.7% (84)	<0.001
Polymyxins	4.9% (60)	4.0% (23)	4.6% (83)	0.415
Vancomycin	3.0% (37)	4.5% (26)	3.5% (63)	0.163
Piperacillin-Tazobactam	2.7% (33)	2.9% (17)	2.8% (50)	0.927
Levofloxacin	2.0% (24)	2.9% (17)	2.3% (41)	0.278
Moxifloxacin	2.1% (25)	2.4% (14)	2.2% (39)	0.766
Others	3.2% (39)	4.0% (23)	3.5% (62)	0.506
Concomitant therapy ^a b^	27.8% (337)	19.2% (112)	25.0% (449)	<0.001
Polymyxins	19.4% (236)	12.4% (72)	17.1% (308)	<0.001
Cefoperazone-Sulbactam	3.0% (37)	1.2% (7)	2.4% (44)	0.028
Imipenem	1.3% (16)	1.2% (7)	1.3% (23)	1
Caspofungin	1.2% (14)	0.3% (2)	0.9% (16)	0.15
Amikacin	0.5% (6)	1.2% (7)	0.7% (13)	0.134
Piperacillin-Tazobactam	0.3% (4)	0.7% (4)	0.4% (8)	0.283
Amphotericin B	0.3% (4)	0.5% (3)	0.4% (7)	0.688
Voriconazole	0.3% (4)	0.3% (2)	0.3% (6)	1
Posaconazole	0.4% (5)	0.2% (1)	0.3% (6)	0.671
Aztreonam	0.2% (2)	0.3% (2)	0.2% (4)	0.599
Others	1.3% (16)	1.2% (7)	1.3% (23)	1
Data presented as percentage (number) or median (IQR), as appropriate.				
a. Total may exceed total number due to receipt of multiple antibiotics.				
b. Concomitant therapy: Antibiotic administered for ≥48 continuous hours while the patient received eravacycline.
P-value was calculated by Utest, χ2/Fisher’s exact test.				

The distribution of infection types reflected the distinct epidemiological profiles of each pathogen. Pneumonia was the predominant manifestation for *A. baumannii*, occurring in 91.7% (1,113/1,214) of cases. The high acuity of these respiratory infections was underscored by the frequent requirement for ventilatory support including mechanical ventilation, endotracheal intubation, and tracheostomy, which was documented in 82.3% (916/1,113) of *A. baumannii* pneumonia patients. Conversely, *K. pneumoniae* infections presented with a broader anatomical distribution. While pneumonia was also common (86.8%, 505/582), a substantial proportion of cases were bloodstream infections (16.7%, 97/582). Among patients with *K. pneumoniae* pneumonia, 67.9% (343/505) required ventilatory support.

Differences were also observed in treatment patterns. Prior to eravacycline initiation, 48.6% of patients with *K. pneumoniae* infections had received pretreatment antimicrobial therapy, with carbapenems (29.6%) and ceftazidime-avibactam (8.1%) being notable exposures. In comparison, the pretreatment exposure rate was 40.2% in the *A. baumannii* group, where carbapenem use was documented in 23.9% of cases and ceftazidime-avibactam in 3.0%. During eravacycline treatment, concomitant antibiotic therapy was employed in 27.8% of *A. baumannii* infections, predominantly with polymyxins (19.4%). This practice was less frequent in *K. pneumoniae* infections, where 19.2% of patients received concomitant therapy, most commonly involving polymyxins (12.4%).

### The antimicrobial susceptibility of isolates

According to the information uploaded by participating hospitals, the antimicrobial susceptibility profiles of ERV (N = 1,070), tigecycline (N = 1,398), imipenem (N = 1,023), meropenem (N = 1,014) and colistin (N = 1,143) were summarized in **Table 2**. Eravacycline demonstrated high activity against both pathogens (overall susceptibility 96.0%, 1,027/1,070), with significantly higher susceptibility for *A. baumannii* versus *K. pneumoniae* (96.9% vs 93.8%; *P* = 0.023). In contrast, tigecycline susceptibility was substantially lower (75.8%, 1,060/1,398).

**Table 2:** Antimicrobial susceptibility of clinical isolates.

	*A. baumannii*	*K. pneumoniae*
Antimicrobial Agents		
Eravacycline	96.85% (738/762)	93.83% (289/308)
Tigecycline	77.71% (753/969)	71.56% (307/429)
Imipenem	5.09% (35/687)	16.07% (54/336)
Meropenem	7.83% (55/702)	15.06% (47/312)
Colistin	92.94% (777/836)	84.36% (259/307)
Eravacycline susceptibility in		
Tigecycline resistant isolates	66.67% (16/24)	82.35% (14/17)
Imipenem resistant isolates	97.74% (390/399)	99.33% (148/149)
Meropenem resistant isolates	96.97% (384/396)	98.57% (138/140)
Colistin resistant isolates	86.67% (26/30)	88% (22/25)

**Figure 1:**
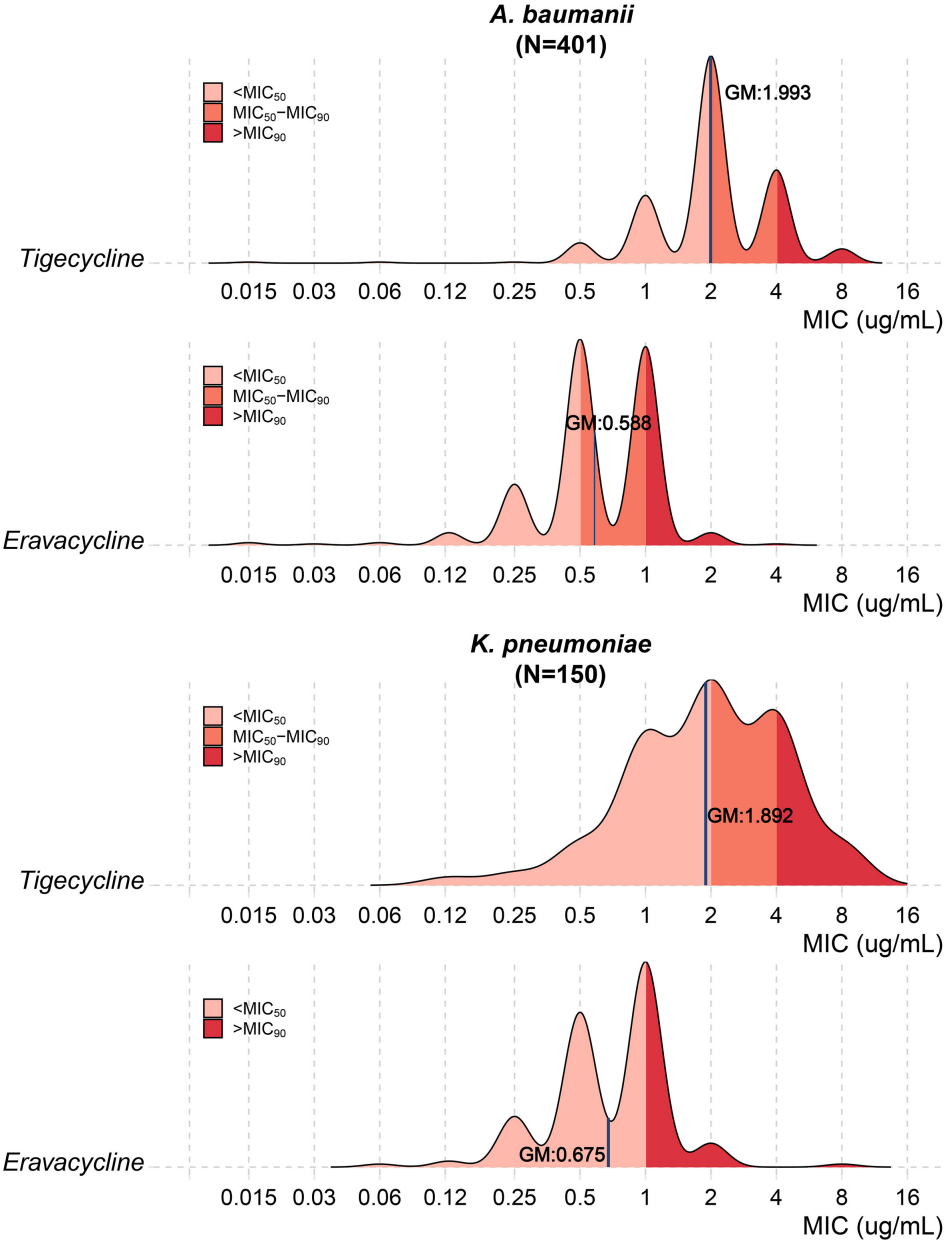
The *in vitro* antimicrobial activity of eravacycline and tigecycline. GM, geometric mean MIC (ug/mL).

Comparative MIC analysis of 551 isolates with MIC for both ERV and tigecycline revealed 4-fold lower geometric mean MICs for eravacycline versus tigecycline against both *A. baumannii* (0.588 vs 1.993 μg/mL; *P* < 0.001) and *K. pneumoniae* (0.675 vs 1.892 μg/mL; *P* < 0.001) (**Figure 1**). Notably, 66.7% (16/24) of tigecycline-resistant *A. baumannii* and 82.4% (14/17) of *K. pneumoniae* isolates remained eravacycline-susceptible (**Table 2**).

Carbapenem non-susceptibility was prevalent: Only 5.1% (35/687) of *A. baumannii* and 16.1% (54/336) of *K. pneumoniae* isolates were imipenem-susceptible. Eravacycline maintained ≥96.9% susceptibility against carbapenem-resistant strains (**Table 2**). Colistin susceptibility was 90.6% (1,036/1,143), with eravacycline retaining activity against 86.7% (26/30) of colistin-resistant *A. baumannii* and 88.0% (22/25) of *K. pneumoniae*.

### Clinical and microbiological outcomes

**Figure 2:**
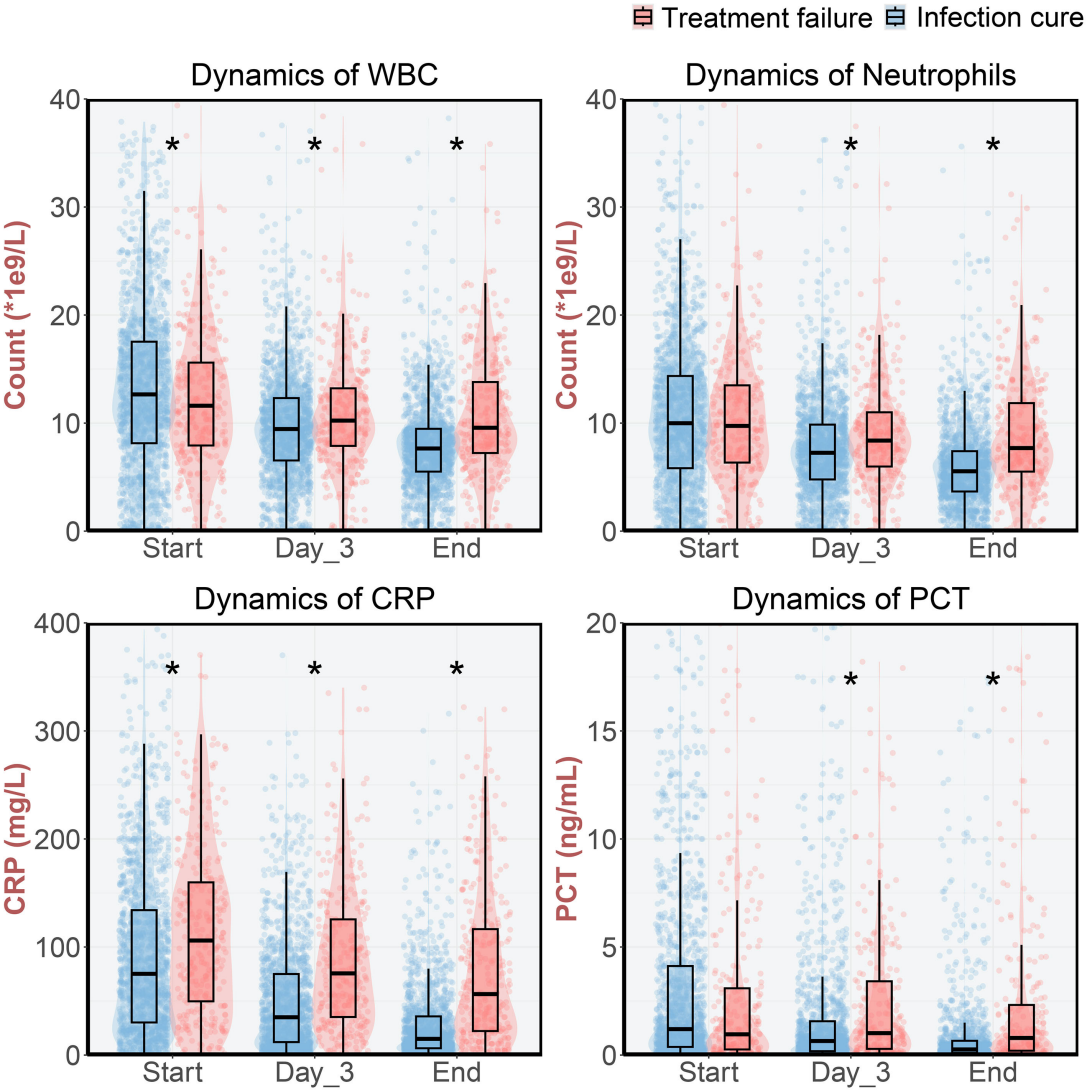
Laboratory test results before eravacycline treatment, at the third day and the end of eravacycline treatment. * indicates p<0.05, compared by Mann-Whitney U-test.

At end of treatment (EOT), microbiological outcomes were assessed in 1,037 patients. Overall microbiological eradication was 76.1% (789/1,037). Microbiological persistence occurred in 9.4% (97/1,037) and relapse in 14.6% (151/1,037) (**Supplementary Table 1**). Among 759 patients without EOT microbiological cultures, clinical cure was achieved in 91.4% (694/759). Composite treatment failure (microbiological persistence/relapse or clinical failure) occurred in 17.4% (313/1,796) of the cohort. Treatment failure was associated with elevated inflammatory markers: Failure cases exhibited significantly higher WBC, neutrophil counts, CRP, and PCT levels at day 3 and EOT versus success cases (*P* < 0.05; **Figure 2**). Monotherapy (75.0%, 1,347/1,796) and concomitant therapy (25.0%, 449/1,796) yielded comparable outcomes (**Supplementary Table 2**).

**Figure 3:**
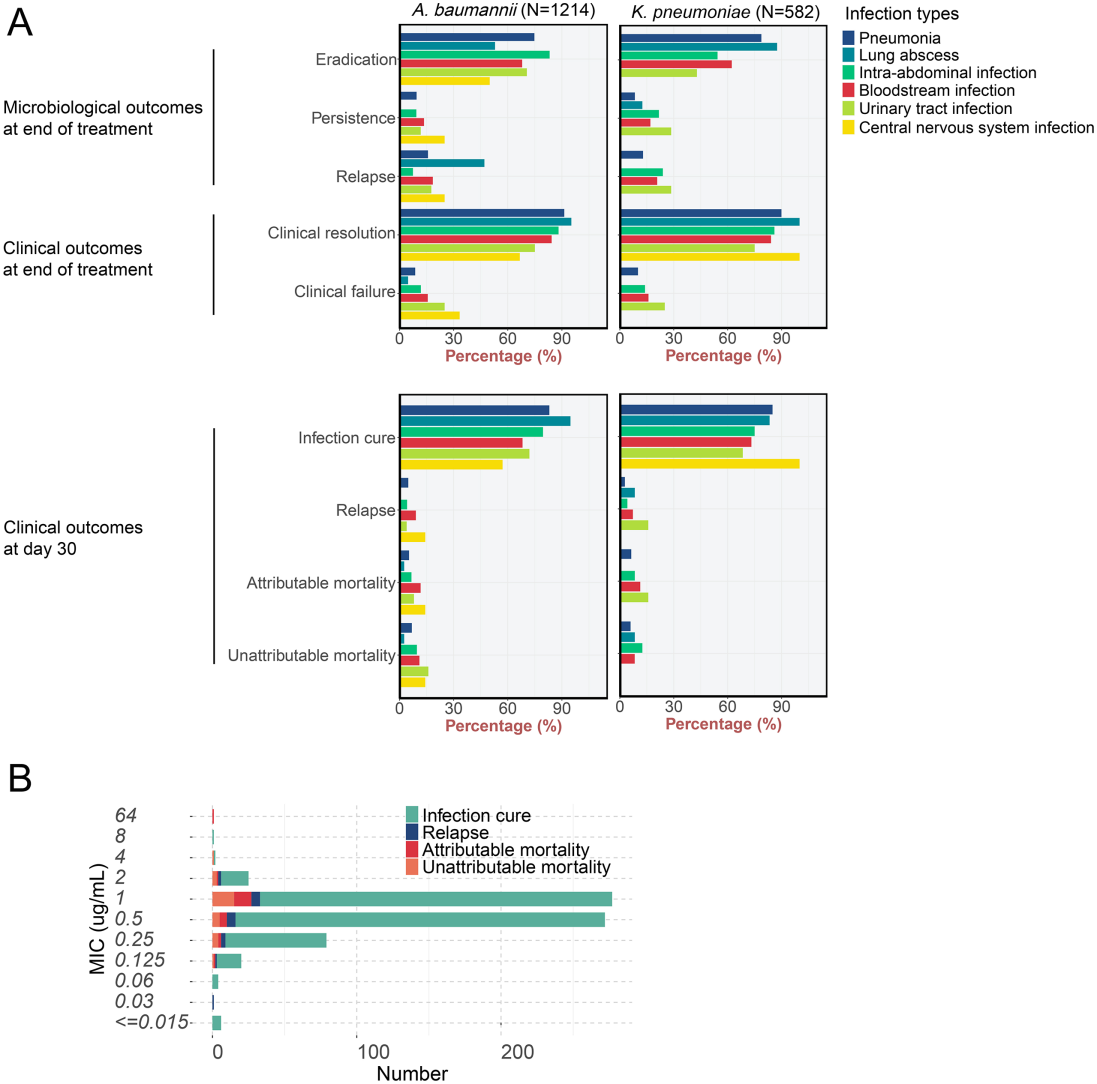
The efficacy of eravacycline treatment in different pathogens **(A)** and isolates with different MICs **(B)**.

The microbiological and clinical outcomes of eravacycline therapy, stratified by the primary infection site, are comprehensively detailed in **Table 3** and **Figure 3A**. The analysis across different infection types revealed distinct patterns of treatment efficacy. Pneumonia, being the most common infection in the cohort, demonstrated favorable outcomes with an overall microbiological eradication rate of 76.0% (723/951) at the end of treatment (EOT). Clinical resolution at EOT was achieved in 90.8% (605/666) of assessable pneumonia patients. By day 30, the infection cure rate for pneumonia was 83.6% (1353/1618), with an attributable mortality of 5.6% (91/1618).

**Table 3:** Microbiological and clinical outcomes by different infection types.

Infection Types and Outcomes	Patients infected with *A. baumannii* (N = 1214)	Patients infected with *K. pneumonia* (N = 582)	Total
Infection Type: Pneumonia			
Microbiological outcomes at end of treatment			
Eradication	497 (74.74%)	226 (78.75%)	723 (75.95%)
Persistence	63 (9.47%)	24 (8.36%)	87 (9.14%)
Relapse with another pathogen	105 (15.79%)	37 (12.89%)	142 (14.92%)
Clinical outcomes at end of treatment			
Clinical resolution	409 (91.29%)	196 (89.91%)	605 (90.84%)
Clinical failure	39 (8.71%)	22 (10.09%)	61 (9.16%)
Clinical outcomes at day 30			
Infection cure	924 (83.02%)	429 (84.95%)	1353 (83.62%)
Relapse	54 (4.85%)	14 (2.77%)	68 (4.20%)
Attributable mortality	59 (5.30%)	32 (6.34%)	91 (5.62%)
Unattributable mortality	76 (6.83%)	30 (5.94%)	106 (6.55%)
Infection Type: Lung abscess			
Microbiological outcomes at end of treatment			
Eradication	9 (52.94%)	7 (87.50%)	16 (64.00%)
Persistence	0 (0.00%)	1 (12.50%)	1 (4.00%)
Relapse with another pathogen	8 (47.06%)	0 (0.00%)	8 (32.00%)
Clinical outcomes at end of treatment			
Clinical resolution	20 (95.24%)	4 (100.00%)	24 (96.00%)
Clinical failure	1 (4.76%)	0 (0.00%)	1 (4.00%)
Clinical outcomes at day 30			
Infection cure	36 (94.74%)	10 (83.33%)	46 (92.00%)
Relapse	0 (0.00%)	1 (8.33%)	1 (2.00%)
Attributable mortality	1 (2.63%)	0 (0.00%)	1 (2.00%)
Unattributable mortality	1 (2.63%)	1 (8.33%)	2 (4.00%)
Infection Type: Intra-abdominal infection			
Microbiological outcomes at end of treatment			
Eradication	89 (83.18%)	25 (54.35%)	114 (74.51%)
Persistence	10 (9.35%)	10 (21.74%)	20 (13.07%)
Relapse with another pathogen	8 (7.48%)	11 (23.91%)	19 (12.42%)
Clinical outcomes at end of treatment			
Clinical resolution	52 (88.14%)	43 (86.00%)	95 (87.16%)
Clinical failure	7 (11.86%)	7 (14.00%)	14 (12.84%)
Clinical outcomes at day 30			
Infection cure	132 (79.52%)	72 (75.00%)	204 (77.86%)
Relapse	7 (4.22%)	4 (4.17%)	11 (4.20%)
Attributable mortality	11 (6.63%)	8 (8.33%)	19 (7.25%)
Unattributable mortality	16 (9.64%)	12 (12.50%)	28 (10.69%)
Infection Type: Bloodstream infection			
Microbiological outcomes at end of treatment			
Eradication	70 (67.96%)	33 (62.26%)	103 (66.03%)
Persistence	14 (13.59%)	9 (16.98%)	23 (14.74%)
Relapse with another pathogen	19 (18.45%)	11 (20.75%)	30 (19.23%)
Clinical outcomes at end of treatment			
Clinical resolution	43 (84.31%)	37 (84.09%)	80 (84.21%)
Clinical failure	8 (15.69%)	7 (15.91%)	15 (15.79%)
Clinical outcomes at day 30			
Infection cure	105 (68.18%)	71 (73.20%)	176 (70.12%)
Relapse	14 (9.09%)	7 (7.22%)	21 (8.37%)
Attributable mortality	18 (11.69%)	11 (11.34%)	29 (11.55%)
Unattributable mortality	17 (11.04%)	8 (8.25%)	25 (9.96%)
Infection Type: Urinary tract infection			
Microbiological outcomes at end of treatment			
Eradication	12 (70.59%)	3 (42.86%)	15 (62.50%)
Persistence	2 (11.76%)	2 (28.57%)	4 (16.67%)
Relapse with another pathogen	3 (17.65%)	2 (28.57%)	5 (20.83%)
Clinical outcomes at end of treatment			
Clinical resolution	6 (75.00%)	9 (75.00%)	15 (75.00%)
Clinical failure	2 (25.00%)	3 (25.00%)	5 (25.00%)
Clinical outcomes at day 30			
Infection cure	18 (72.00%)	13 (68.42%)	31 (70.45%)
Relapse	1 (4.00%)	3 (15.79%)	4 (9.09%)
Attributable mortality	2 (8.00%)	3 (15.79%)	5 (11.36%)
Unattributable mortality	4 (16.00%)	0 (0.00%)	4 (9.09%)
Infection Type: Central nervous system infection			
Microbiological outcomes at end of treatment			
Eradication	2 (50.00%)	0 (0.00%)	2 (50.00%)
Persistence	1 (25.00%)	0 (0.00%)	1 (25.00%)
Relapse with another pathogen	1 (25.00%)	0 (0.00%)	1 (25.00%)
Clinical outcomes at end of treatment			
Clinical resolution	2 (66.67%)	1 (100.00%)	3 (75.00%)
Clinical failure	1 (33.33%)	0 (0.00%)	1 (25.00%)
Clinical outcomes at day 30			
Infection cure	4 (57.14%)	1 (100.00%)	5 (62.50%)
Relapse	1 (14.29%)	0 (0.00%)	1 (12.50%)
Attributable mortality	1 (14.29%)	0 (0.00%)	1 (12.50%)
Unattributable mortality	1 (14.29%)	0 (0.00%)	1 (12.50%)

For other infection types, the overall EOT microbiological eradication rates were 64.0% (16/25) for lung abscess, 74.5% (114/153) for intra-abdominal infection, 66.0% (103/156) for bloodstream infection, 62.5% (15/24) for urinary tract infection, and 50.0% (2/4) for central nervous system infection. The corresponding day 30 infection cure rates for these infection types were 92.0% (46/50), 77.9% (204/262), 70.1% (176/251), 70.5% (31/44), and 62.5% (5/8), respectively. Across all infection types, clinical resolution rates at the end of treatment were consistently high, ranging from 75.0% to 96.0%. The 30-day attributable mortality was lowest in pneumonia (5.6%) and lung abscess (2.0%), and highest in bloodstream infections (11.6%) and central nervous system infections (12.5%).

### Factors associated with treatment failure

Among 1,070 cases with available ERV susceptibility data, infections caused by non-susceptible strains (MIC >1 μg/mL) showed a higher rate of treatment failure at the end of treatment (23.26%, 10/43) compared to susceptible strains (12.95%, 133/1027), though this difference did not reach statistical significance (p=0.086) (**Table 4** and **Figure 3B**). Multivariable logistic regression analysis (**Supplementary Table 3**) identified several independent predictors of early treatment failure. Higher baseline white blood cell (WBC) count was associated with reduced risk (OR 0.95, 95% CI: 0.92–0.98, p<0.001), whereas elevated inflammatory markers, including baseline CRP (OR 1.00, 95% CI: 0.99–1.00, p=0.048) and CRP after 3 days of treatment (OR 1.01, 95% CI: 1.01–1.01, p<0.001), significantly increased the risk of failure. Increased neutrophil counts at day 3 also predicted poorer outcomes (OR 1.04, 95% CI: 1.01–1.07, p=0.018). Clinical interventions influenced outcomes substantially: central venous catheterization (OR 2.15, 95% CI: 1.18–3.90, p=0.012), tracheostomy (OR 2.86, 95% CI: 1.75–4.66, p<0.001), and other indwelling catheters (OR 2.49, 95% CI: 1.27–4.86, p=0.008) were associated with higher failure rates. In contrast, mechanical ventilation (OR 0.52, 95% CI: 0.29–0.93, p=0.029) and arterial catheterization (OR 0.58, 95% CI: 0.34–0.99, p=0.048) appeared protective.

**Table 4:** Microbiological and clinical outcomes of patients infected with susceptible or non-susceptible strains.

Outcomes	Infection of strains with MIC > 1 (N = 43)	Infection of strains with MIC ≤ 1 (N = 1027)	*P*
Microbiological outcomes at end of treatment			
Eradication	16 (66.67%)	488 (80.4%)	0.08
Persistence	1 (4.17%)	43 (7.08%)	
Relapse with another pathogen	7 (29.17%)	76 (12.52%)	
Clinical outcomes at end of treatment			
Clinical resolution	17 (89.47%)	406 (96.67%)	0.15
Clinical failure	2 (10.53%)	14 (3.33%)	
Clinical outcomes at day 30			
Infection cure	32 (74.42%)	901 (87.73%)	0.04
Relapse	2 (4.65%)	26 (2.53%)	
Attributable mortality	4 (9.3%)	40 (3.89%)	
Unattributable mortality	5 (11.63%)	60 (5.84%)	
P-value was calculated byχ2/Fisher’s exact test.			

At day 30, the overall cure rate was 83.57% (1501/1,796). Relapse occurred in 4.34% (78/1,796) of cases, and overall mortality was 12.08% (217/1,796), with 5.4% (97/1,796) attributed directly to infection. Infections caused by non-susceptible isolates were associated with significantly lower cure rates (74.4% *vs*. 87.7%, p=0.04) (**Table 4**). This association was attenuated and lost statistical independence in the adjusted model (OR 2.73, 95%CI: 0.88-8.45, p=0.082). Multivariable analysis (**Supplementary Table 4**) indicated that elevated CRP levels at the end of treatment, was the strongest predictor of 30-day failure (OR 1.03, 95% CI: 1.02–1.04, p<0.001). Bloodstream infection (OR 2.41, 95% CI: 1.18–4.93, p=0.016) and sepsis (OR 1.94, 95% CI: 1.01–3.69, p=0.045) were also independent risk factors, nearly doubling the odds of failure. Invasive procedures further modulated outcomes: endotracheal intubation increased risk (OR 2.65, 95% CI: 1.21–5.81, p=0.015), whereas mechanical ventilation was strongly protective (OR 0.27, 95% CI: 0.13–0.58, p<0.001).

### Adverse events

Adverse events (AEs) occurred in 41 patients (2.28%, 41/1,796). The most frequent AEs were gastrointestinal disorders (1.11%, 20/1,796) and hepatotoxicity (0.56%, 10/1,796). One patient experienced a severe AE resulting in acute liver injury.

## Discussion

Our study presents the largest real-world, observational analysis of clinical and microbiological outcomes for patients treated with ERV for infection caused by *A. baumannii* or *K. pneumoniae*. Most of patients were from ICU (57.41%) and department of hematology (11.08%), with a median age of 62 (50–74) and various underlying medical conditions like hematological system diseases and immunosuppressant usage, as well as medical ventilation. Our cohort represents patients with difficult-to-treat infection in a real-world hospital setting.

The pharmacokinetic (PK) and pharmacodynamic (PD) profile of eravacycline provides a crucial lens through which to interpret our clinical outcomes. Eravacycline is characterized by a high volume of distribution (ranging from ~184 to 320 L in human studies) (Connors et al., 2014; Zhanel et al., 2016), which signifies extensive penetration into body tissues. This property is a key asset, likely contributing to the favorable clinical and microbiological outcomes observed in our cohorts with tissue-based infections such as pneumonia and intra-abdominal infections, and may also support its efficacy in bloodstream infections by ensuring adequate tissue sanctuary site penetration. Conversely, its PK profile also explains the observed trends in urinary tract infections (UTIs). Eravacycline undergoes minimal renal excretion, with studies reporting very low urinary recovery (e.g., ~2.5% in rats) and the majority of the drug being eliminated via the fecal route (Tan et al., 2020). The low urinary concentrations achieved are pharmacologically suboptimal for eradicating pathogens in the urinary tract. Consequently, the numerically lower eradication rate we observed for UTIs (62.5%), though not powered for statistical significance, is consistent with its intrinsic PK properties.

*A. baumannii* usually exhibits a high drug resistance rate, and is one of the most challenging pathogens in the health care setting (Ayoub Moubareck and Hammoudi Halat, 2020). The IGNITE1 trial (Solomkin et al., 2017) demonstrated clinical cure with ERV in 8/8 patients infected with *A. baumannii*, of which 2 of them were confirmed CRAB. Then, the IGNITE4 trial (Solomkin et al., 2019) showed 100% (5/5) clinical cure rate for patients infected with *A. baumannii*. Alosaimy et al. (Alosaimy et al., 2022). conducted a retrospective analysis on 46 patients treated with ERV for *A. baumannii* infections (69.5% of them were CRAB) and observed a 30-day mortality of 23.9%. Scott et al. (Scott et al., 2022) analyzed 27 patients receiving ERV for pneumonia with *A. baumannii*. However, they observed higher 30-day mortality (33% vs 15%; p = 0.048) and lower microbiologic cure rate (17% vs 59%; p = 0.004) compared with control group. Few studies had focused on ERV treatment of *K. pneumoniae* infection. Study of Hise et al. (Van Hise et al., 2020) included 3 patients infected with *K. pneumoniae* and clinical failure occurred in one of them. In a real-world, observational study in USA, Kunz, et al. (Kunz Coyne et al., 2024). reported clinical success rate of 75.7% (n = 315/416) in patients infected with Enterobacterales spp., Enterococci spp. and Acinetobacter spp. Supported by clinical and microbiological data of 1214 A*. baumannii* infected patients with various underlying medical conditions, we observed an eradication rate of 75.63% and a 30-day infection cure rate of 83.11%. Similar trend was also noticed in 582 K*. pneumoniae* infected patients, with an eradication rate of 77.09% and a 30-day infection cure rate of 84.54%. More importantly, many isolates in our study were resistant to imipenem or meropenem, and around 97% of them were susceptible to ERV. Consistently, previous *in vitro* studies had underscored the potency of ERV against carbapenem-resistant pathogens (Seifert et al., 2018). We believe ERV provides a new option for carbapenem-resistant strains.

As a new tetracycline antibacterial agents, ERV seems to have better performance that tigecycline. Previous comparison study of ERV with tigecycline show that in terms of efficacy, ERV has a higher clinical response rate and a significantly better microbiological response rate than tigecycline (Meng et al., 2022). ERV has a 2- to 4-fold lower MIC_90_ than tigecycline against common gram-negative bacteria in both the overall and multidrug-resistant populations (Zhao et al., 2019; Morrissey et al., 2020). Similarly, we also observed 4-fold lower MIC_90_ in ERV than tigecycline against *A. baumannii* or *K. pneumoniae*. This superior *in vitro* activity appears to translate into survival benefits in clinical practice; a recent real-world study by Guo et al (Guo et al., 2025). involving ICU patients with CRAB pneumonia reported a notably lower 30-day mortality rate in the eravacycline group (15.2%) compared to the tigecycline group (25.0%), reinforcing the utility of eravacycline in high-acuity settings. Besides that, it has been suggested that ERV might be less likely than tigecycline to cause nausea (Scott, 2019). The safety profile of eravacycline in our large cohort was consistent with prior reports, with gastrointestinal events being the most common adverse effect (Van Hise et al., 2020; Eljaaly et al., 2021). The low incidence of hepatotoxicity (0.56%) is reassuring.

Previous meta-analysis did not offer a thorough analysis of the clinical outcomes associated with ERV monotherapy or combination therapy (Eljaaly et al., 2021; Chen et al., 2024). In our study, ERV was used in monotherapy in 75% of the cases. Amid cases taken combination therapy, 68.60% of them used ERV in combination with polymyxin. However, different ERV regimens had no influence on the microbiological and clinical outcomes.

The multivariable analyses further revealed that persistent inflammation—particularly elevated CRP levels at both early and late treatment phases—was a dominant predictor of treatment failure. This association underscores the diagnostic value of inflammatory kinetics; as previously observed in severe bloodstream infections and ventilator-associated pneumonia, a CRP ratio greater than 0.58 on the fourth day of therapy is a strong predictor of poor outcomes, reflecting an inadequate host or therapeutic response (Póvoa et al., 2005; Póvoa et al., 2017). In successful cases, CRP typically declines to approximately 47% of initial levels by day 4, whereas it remains nearly unchanged in non-responders (Póvoa et al., 2017). Furthermore, the higher failure rates observed in patients with sepsis may be linked to altered pharmacokinetics. Sepsis-induced capillary leak and aggressive fluid resuscitation can significantly increase the volume of distribution, potentially leading to subtherapeutic serum concentrations of drugs with high tissue distribution like eravacycline (Blot et al., 2014; Shah et al., 2015). The increased risks also linked to central venous catheterization and other indwelling devices emphasize the iatrogenic contributions to treatment failure, likely through biofilm formation or secondary infections. The observation that non-susceptibility to ERV remained a strong predictor in univariate analysis but was attenuated in multivariable models suggests that its effect may be mediated or confounded by other patient factors, such as underlying comorbidities or inflammatory status.

Data should be interpreted with caution because of the retrospective nature of the study. As a retrospective observational study without a randomized control group, we cannot definitively establish the superiority of eravacycline over other standard therapies; our findings should be interpreted as reflecting real-world clinical utility rather than controlled comparative efficacy. Furthermore, this study was conducted exclusively within China, and the results may reflect local resistance patterns and clinical practices. Therefore, global multicenter studies are needed to confirm these findings across diverse populations. Not all ERV MICs data were collected. Limited carbapenem MICs data also lead to an unprecise proportion of carbapenem-resistant strains. Information on source control measures (e.g., drainage or debridement), a critical confounder for clinical efficacy in infections such as lung abscesses and intra-abdominal infections, was not consistently available across all centers in this retrospective study and could not be analyzed. Future prospective studies should prioritize the standardized collection of this data. Furthermore, this study focused exclusively on *A. baumannii* and *K. pneumoniae*. Expanding future research to include other difficult-to-treat *Enterobacterales* would be valuable to further establish eravacycline’s role across a broader spectrum of Gram-negative infections. Finally, the molecular mechanisms underlying eravacycline non-susceptibility were not investigated in this study. Future work should include genomic analysis to characterize these resistance pathways.

The present study is the largest report of eravacycline use in China hospitals to date, ERV demonstrated promising efficacy in treating patients with infections of *A. baumannii* or *K. pneumoniae*, especially carbapenem-resistant strains.

## Data Availability

The original contributions presented in the study are included in the article/**Supplementary Material**. Further inquiries can be directed to the corresponding authors.

## References

[B1] Alosaimy S. Abdul-Mutakabbir J. C. Kebriaei R. Jorgensen S. C. J. Rybak M. J. (2020a). Evaluation of eravacycline: A novel fluorocycline. Pharmacotherapy 40, 221–238. doi: 10.1002/phar.2366, PMID: 31944332

[B2] Alosaimy S. Molina K. C. Claeys K. C. Andrade J. Truong J. King M. A. . (2020b). Early experience with eravacycline for complicated infections. Open Forum Infect. Dis. 7, ofaa071. doi: 10.1093/ofid/ofaa071, PMID: 32411809 PMC7210802

[B3] Alosaimy S. Morrisette T. Lagnf A. M. Rojas L. M. King M. A. Pullinger B. M. . (2022). Clinical outcomes of eravacycline in patients treated predominately for carbapenem-resistant acinetobacter baumannii. Microbiol. Spectr. 10, e0047922. doi: 10.1128/spectrum.00479-22, PMID: 36190427 PMC9602915

[B4] Ayoub Moubareck C. Hammoudi Halat D. (2020). Insights into Acinetobacter baumannii: A Review of Microbiological, Virulence, and Resistance Traits in a Threatening Nosocomial Pathogen. Antibiotics (Basel) 9, 119. doi: 10.3390/antibiotics9030119, PMID: 32178356 PMC7148516

[B5] Blot S. I. Pea F. Lipman J. (2014). The effect of pathophysiology on pharmacokinetics in the critically ill patient--concepts appraised by the example of antimicrobial agents. Adv. Drug Delivery Rev. 77, 3–11. doi: 10.1016/j.addr.2014.07.006, PMID: 25038549

[B6] Chen Z. Sun W. Chi Y. Liang B. Cai Y. (2024). Efficacy and safety of eravacycline (ERV) in treating infections caused by Gram-negative pathogens: a systematic review and meta-analysis. Expert Rev. Anti Infect. Ther. 22, 867–875. doi: 10.1080/14787210.2024.2397663, PMID: 39258866

[B7] Connors K. P. Housman S. T. Pope J. S. Russomanno J. Salerno E. Shore E. . (2014). Phase I, open-label, safety and pharmacokinetic study to assess bronchopulmonary disposition of intravenous eravacycline in healthy men and women. Antimicrob. Agents Chemother. 58, 2113–2118. doi: 10.1128/AAC.02036-13, PMID: 24468780 PMC4023791

[B8] Eljaaly K. Ortwine J. K. Shaikhomer M. Almangour T. A. Bassetti M. (2021). Efficacy and safety of eravacycline: A meta-analysis. J. Glob Antimicrob. Resist. 24, 424–428. doi: 10.1016/j.jgar.2021.02.009, PMID: 33621690

[B9] Guo Q. Wei Y. Zhao Q. Zhang W. Lv P. Zhou Z. . (2025). Efficacy and safety of eravacycline combination therapy for carbapenem-resistant acinetobacter baumannii pneumonia in ICU patients: A retrospective study. Infect. Drug Resist. 18, 3013–3021. doi: 10.2147/IDR.S515207, PMID: 40548167 PMC12182229

[B10] Harding C. M. Hennon S. W. Feldman M. F. (2018). Uncovering the mechanisms of Acinetobacter baumannii virulence. Nat. Rev. Microbiol. 16, 91–102. doi: 10.1038/nrmicro.2017.148, PMID: 29249812 PMC6571207

[B11] Kunz Coyne A. J. Alosaimy S. Lucas K. Lagnf A. M. Morrisette T. Molina K. C. . (2024). Eravacycline, the first four years: health outcomes and tolerability data for 19 hospitals in 5 U.S. Reg 2018 to 2022. Microbiol. Spectr. 12, e0235123. doi: 10.1128/spectrum.02351-23, PMID: 38018984 PMC10782980

[B12] Lan S. H. Chang S. P. Lai C. C. Lu L. C. Chao C. M. (2019). The efficacy and safety of eravacycline in the treatment of complicated intra-abdominal infections: A systemic review and meta-analysis of randomized controlled trials. J. Clin. Med. 8, 866. doi: 10.3390/jcm8060866, PMID: 31212991 PMC6617347

[B13] Lei T. Y. Liao B. B. Yang L. R. Wang Y. Chen X. B. (2024). Hypervirulent and carbapenem-resistant Klebsiella pneumoniae: A global public health threat. Microbiol. Res. 288, 127839. doi: 10.1016/j.micres.2024.127839, PMID: 39141971

[B14] Marí-Almirall M. Cosgaya C. Higgins P. G. Van Assche A. Telli M. Huys G. . (2017). MALDI-TOF/MS identification of species from the Acinetobacter baumannii (Ab) group revisited: inclusion of the novel A. seifertii and A. dijkshoorniae species. Clin. Microbiol. Infect. 23, 210.e211–210.e219. doi: 10.1016/j.cmi.2016.11.020, PMID: 27919649

[B15] Meng R. Guan X. Sun L. Fei Z. Li Y. Luo M. . (2022). The efficacy and safety of eravacycline compared with current clinically common antibiotics in the treatment of adults with complicated intra-abdominal infections: A Bayesian network meta-analysis. Front. Med. (Laus) 9, 935343. doi: 10.3389/fmed.2022.935343, PMID: 36186801 PMC9524542

[B16] Morrissey I. Olesky M. Hawser S. Lob S. H. Karlowsky J. A. Corey G. R. . (2020). *In vitro* activity of eravacycline against gram-negative bacilli isolated in clinical laboratories worldwide from 2013 to 2017. Antimicrob. Agents Chemother. 64, e01699-19. doi: 10.1128/AAC.01699-19, PMID: 31843999 PMC7038303

[B17] Póvoa P. Coelho L. Almeida E. Fernandes A. Mealha R. Moreira P. . (2005). Pilot study evaluating C-reactive protein levels in the assessment of response to treatment of severe bloodstream infection. Clin. Infect. Dis. 40, 1855–1857. doi: 10.1086/430382, PMID: 15909277

[B18] Póvoa P. Martin-Loeches I. Ramirez P. Bos L. D. Esperatti M. Silvestre J. . (2017). Biomarkers kinetics in the assessment of ventilator-associated pneumonia response to antibiotics - results from the BioVAP study. J. Crit. Care 41, 91–97. doi: 10.1016/j.jcrc.2017.05.007, PMID: 28502892

[B19] Scott L. J. (2019). Eravacycline: A review in complicated intra-abdominal infections. Drugs 79, 315–324. doi: 10.1007/s40265-019-01067-3, PMID: 30783960 PMC6505493

[B20] Scott C. J. Zhu E. Jayakumar R. A. Shan G. Viswesh V. (2022). Efficacy of eravacycline versus best previously available therapy for adults with pneumonia due to difficult-to-treat resistant (DTR) acinetobacter baumannii. Ann. Pharmacother. 56, 1299–1307. doi: 10.1177/10600280221085551, PMID: 35511209

[B21] Seifert H. Stefanik D. Sutcliffe J. A. Higgins P. G. (2018). *In-vitro* activity of the novel fluorocycline eravacycline against carbapenem non-susceptible Acinetobacter baumannii. Int. J. Antimicrob. Agents 51, 62–64. doi: 10.1016/j.ijantimicag.2017.06.022, PMID: 28705668

[B22] Shah S. Barton G. Fischer A. (2015). Pharmacokinetic considerations and dosing strategies of antibiotics in the critically ill patient. J. Intensive Care Soc. 16, 147–153. doi: 10.1177/1751143714564816, PMID: 28979397 PMC5606477

[B23] Solomkin J. Evans D. Slepavicius A. Lee P. Marsh A. Tsai L. . (2017). Assessing the efficacy and safety of eravacycline vs ertapenem in complicated intra-abdominal infections in the investigating gram-negative infections treated with eravacycline (IGNITE 1) trial: A randomized clinical trial. JAMA Surg. 152, 224–232. doi: 10.1001/jamasurg.2016.4237, PMID: 27851857

[B24] Solomkin J. S. Gardovskis J. Lawrence K. Montravers P. Sway A. Evans D. . (2019). IGNITE4: results of a phase 3, randomized, multicenter, prospective trial of eravacycline vs meropenem in the treatment of complicated intraabdominal infections. Clin. Infect. Dis. 69, 921–929. doi: 10.1093/cid/ciy1029, PMID: 30561562 PMC6735687

[B25] Sutcliffe J. A. O’Brien W. Fyfe C. Grossman T. H. (2013). Antibacterial activity of eravacycline (TP-434), a novel fluorocycline, against hospital and community pathogens. Antimicrob. Agents Chemother. 57, 5548–5558. doi: 10.1128/AAC.01288-13, PMID: 23979750 PMC3811277

[B26] Tan X. Zhang M. Liu Q. Wang P. Zhou T. Zhu Y. . (2020). Nonclinical pharmacokinetics, protein binding, and elimination of KBP-7072, an aminomethylcycline antibiotic, in animal models. Antimicrob. Agents Chemother. 64, e00488-20. doi: 10.1128/AAC.00488-20, PMID: 32229494 PMC7269484

[B27] Van Hise N. Petrak R. M. Skorodin N. C. Fliegelman R. M. Anderson M. Didwania V. . (2020). A real-world assessment of clinical outcomes and safety of eravacycline: A novel fluorocycline. Infect. Dis. Ther. 9, 1017–1028. doi: 10.1007/s40121-020-00351-0, PMID: 33063176 PMC7680490

[B28] Zhanel G. G. Cheung D. Adam H. Zelenitsky S. Golden A. Schweizer F. . (2016). Review of eravacycline, a novel fluorocycline antibacterial agent. Drugs 76, 567–588. doi: 10.1007/s40265-016-0545-8, PMID: 26863149

[B29] Zhao C. Wang X. Zhang Y. Wang R. Wang Q. Li H. . (2019). *In vitro* activities of Eravacycline against 336 isolates collected from 2012 to 2016 from 11 teaching hospitals in China. BMC Infect. Dis. 19, 508. doi: 10.1186/s12879-019-4093-1, PMID: 31182038 PMC6558774

